# The association of *CASC16* variants with breast Cancer risk in a northwest Chinese female population

**DOI:** 10.1186/s10020-020-0137-7

**Published:** 2020-01-29

**Authors:** Xiaoxiao Zuo, Huanhuan Wang, Yin Mi, Yue Zhang, Xiaofei Wang, Ya Yang, Suna Zhai

**Affiliations:** Department of Radiation Oncology, First Affiliated Hospital of Zhengzhou University, #2 East Jianshe Road, Zhengzhou, 450000 Henan China

**Keywords:** Breast cancer, *CSAC16*, Polymorphism, Susceptibility

## Abstract

**Purpose:**

Genetic variants play a critical role in the development of breast cancer. This investigation aimed to explore the association between *CASC16* polymorphisms and breast cancer susceptibility.

**Methods:**

We conducted a case-control study of 681 patients and 680 healthy individuals to investigate the correlation of five SNPs with breast cancer in a Northwest Chinese female population. Odds ratios (OR) and 95% confidence intervals (CIs) were used to assess the association.

**Results:**

Our study found that rs4784227 and rs12922061 were significantly related to an increased susceptibility to breast cancer (OR 1.22, *p* = 0.022; OR 1.21, *p* = 0.026). While rs3803662 was a protective role in breast cancer risk (OR 0.69, *p* = 0.042). Stratified analyses indicated that rs4784227 and rs12922061 would increase breast cancer susceptibility at age >  50 years. Rs3803662 was a reduced factor of breast cancer risk by age ≤ 50 years. Rs4784227 was significantly increased risk of breast cancer in stage III/IV. The rs45544231 and rs3112612 had a protective effect on breast cancer with tumor size > 2 cm. Rs4784227 and rs12922061 could enhance breast cancer risk in lymph node metastasis positive individuals. *CASC16* rs12922061 and rs4784227 polymorphisms correlated with an increased risk of breast cancer in BMI >  24 kg/m^2^. Haplotype analyses revealed that G_rs45544231_ T_rs12922061_ A_rs3112612_ and G_rs45544231_ C_rs12922061_ A_rs3112612_ haplotypes decreased breast cancer risk.

**Conclusion:**

Our study revealed that *CASC16* genetic variants were significantly related to breast cancer susceptibility, which might give scientific evidence for exploring the molecular mechanism of breast cancer.

## Introduction

Breast cancer (BC) is one of the common malignant tumors in women (Torre et al. 2017) and the 2nd leading cause of cancer death among females in China (Chen et al. 2016a). The China National Cancer Centre recently reported that the incidence of breast cancer is 7.33% in China, of which 6.29% is in the northwest. Breast cancer showed a high mortality (2.70%) and the highest incidence (5.70%) rates in women of Northwest China. As of 2014, the newly increased incidence rates were 25.33, 24.47, and 11.28% among those aged 15–44 years, 45–59 years and 60–79 years, respectively (Wanqing et al. 2014; F B, et al. 2018; Wan-qing et al. 2019). As a kind of multifactorial disease, BC is due to complex non-genetic and genetic factors (Rudolph 2016). Although non-genetic factors such as age, age of menarche, body mass index (BMI), procreative, and menstrual history were associated with an increased susceptibility to breast cancer (Anderson et al. 2004; Islam et al. 2013; Nelson et al. 2012; Zarco et al. 2012). Many recent studies have established that genetic factor also had a vital role in progression of breast cancer (Bray et al. 2013; Sehrawat et al. 2011; Ruiz-Narvaez et al. 2013; Han et al. 2011), and there were 27% of the breast cancer risk influenced by genetic variants (Lichtenstein et al. 2000). In addition, a number of genes including *BRCA1*, *BRCA2*, *PTEN*, *TP53*, *CYP17* and other different genes have demonstrated that their polymorphisms were associated with risk of breast cancer (Nelson et al. 2012; Liao et al. 2018; Walsh and King 2007; Han et al. 2016a; Wang et al. 2016; Lilyquist et al. 2018; He et al. 2014; Chen et al. 2016b; Yang et al. 2018).

Cancer-susceptibility candidate 16 gene (*CASC16*), also termed *LOC643714*, is a kind of long non-protein coding RNA and located at chromosome 16q12.1. Data from one study showed that *CASC16* gene had a higher expression in breast cancer cells compared with normal cells (Han et al. 2016b). Furthermore, several studies had revealed a correlation between *LOC643714* gene and BC (He et al. 2014; Ruiz-Narvaez et al. 2010; Low et al. 2013), but the functions of this gene are still unknown. Liao et al. found that rs12922061 polymorphism of the *CASC16* gene was significantly increased susceptibility to breast cancer in southern China population (Liao et al. 2018). And the rs3803662 and rs12922061 also could increase the risk of breast cancer in a Japanese population (Low et al. 2013). However, another study indicated that rs4784227 of *LOC643714* could improve BC risk, but rs3803662 and rs3112612 haven’t observed a significant association in a southern Chinese population (He et al. 2014). The rs3803662 of *LOC643714* also had no significant association with BC risk in African-American women (Ruiz-Narvaez et al. 2010). These differences in the previous results may be due to the race, geographical location, lifestyle, and environmental exposure in specific Chinese population, which may be resulted in differences in the frequencies of genetic polymorphisms. As we all known, the Han Chinese population exhibits a complicated substructure, because the genes of northern China differ greatly from those of Southern China. However, the previous studies mainly focused on rs3803662, rs12922061, and rs3112612 polymorphisms in *CASC16* association with breast cancer risk in a Southern Chinese population. The correlation between these three SNPs and breast cancer hadn’t been identified in the Northwest Chinese population.

In this case-control study, we selected five SNPs (rs3803662, rs4784227, rs45544231, rs12922061, and rs3112612) in the *CASC16* gene according to the previous studies and the 1000 genomes project. We further investigated the association between *CASC16* genetic variants and BC susceptibility in a Northwest Chinese female population. Our findings would give available information for prevention and management of breast cancer.

## Materials and methods

### Study population

In this present case-control study, 681 unrelated Chinese female breast cancer patients and 680 healthy subjects were recruited from the Shaanxi Provincial Cancer Hospital. All patients were newly diagnosed with histological examination and confirmed to be BC. Patients with a history of autoimmunity, secondary tumors, severe infections diseases, other types of cancer and family history of any cancers included breast cancer were excluded. Healthy individuals were matched with the case subjects based on age and ethnicity, who were randomly selected from the cancer-free female population with a routine health examination in the same hospital. Controls with the family history of any cancers were excluded. Each study participant was informed the purpose of the sample collection and their written consent were obtained. The participants’ basic information were obtained from the patients or their medical records including age, ethnicity, place of residence, tutor position, lymph node metastasis status, clinical stage, tumor size, estrogenic receptor (ER), progesterone receptor (PR) status, menopausal status, procreative times, age of menarche, and body mass index (BMI). All experiments were carried out depending on the guideline of Helsinki’s declaration and our study were approved by the ethics committee of the Shaanxi Provincial Cancer Hospital.

### Selection of SNPs and genotype analysis

We selected five polymorphisms of *CASC16* in the present study. Of the five SNPs, three polymorphisms (rs3803662, rs12922061, and rs3112612) were chosen basing upon the published papers which they reported that these SNPs might be related to breast cancer susceptibility (He et al. 2014). While rs4784227 and rs45544231 were obtained from the 1000 Genomes Project with a minor allele frequency (MAF) > 5% for further genotype. We extracted genomic DNA from peripheral blood samples from the study participants using a blood genomic DNA extraction kit (GoldMag, Xi’an, China). NanoDrop 2000C spectrophotometer (Thermo Scientific, Waltham, USA) were implemented to check purity and concentration of the genomic DNA and then kept at − 20 °C for further analysis. We used Agena Bioscience Assay Design Suite V2.0 software (https://agenacx.com/online-tools/) to design PCR primers. SNP genotype was identified by Agena MassARRAY iPLEX platform, and Agena Bioscience TYPER version 4.0 software was used to manage and analyze the data (Xia et al. 2014; Zhou et al. 2015). To validate the genotype results, 10% of samples were randomly selected, and genotypes showed 100% concordance for all SNPs according to Sanger sequencing.

### Statistical analysis

The differences in demographic characteristics between the case and control group were analyzed by continuous variable independent sample t-test and category variable Pearson’s chi-square test. Hardy–Weinberg equilibrium (HWE) of each SNP was tested by chi-squared test to assess genotype frequencies in controls. Comparisons of distribution in SNP allele and genotype frequencies between case and control were checked by a Pearson chi-squared test or Fisher′s exact test. The association between *CASC16* SNPs and BC susceptibility were assessed by computing odds ratios (ORs) and 95% confidence intervals (CIs) in five inheritance models (allele, co-dominant, dominant, recessive, and log-additive) using logistic regression analysis with or without adjustment for age or BMI. Linkage disequilibrium (LD) was constructed by Haploview V4.2 software and haplotype was analyzed by logistic regression. Besides, we also evaluated the relationship between *CASC16* polymorphisms and BC patient subgroups with stratification analyses. All statistical analyses were performed using SPSS version 17.0 software (IBM Analytics, Chicago, IL) and PLINK software. All statistical tests were two-tailed and *p-*value < 0.05 was considered statistical significance.

## Results

### Characteristics of the study population

The basic information of the study subjects was summarized in Table 1. The average ages were 50.58 ± 9.84 years in cases and 50.63 ± 9.71 years in controls. There was no significant difference in age between the case and control group (*p* = 0.930).

**Table 1:** Characteristic of breast cancer patients and health control individuals

Variables	Cases (*n* = 681)	Controls (*n* = 680)	*p*
Age, years (mean ± SD)^a^	50.58 ± 9.84	50.63 ± 9.71	0.930
> 50	345 (51%)	344 (51%)	
≤ 50	336 (49%)	336 (49%)	
Tumor position			
Left	274 (40%)		
Right	288 (42%)		
Missing	119 (18%)		
LN metastasis			
Node-positive	323 (47%)		
Node-negative	331 (49%)		
Missing	27 (4%)		
Clinical stage			
III/IV	150 (22%)		
I/II	321 (47%)		
Missing	210 (31%)		
Tumor size			
> 2 cm	409 (60%)		
≤ 2 cm	139 (20%)		
Missing	133 (20%)		
PR			
Positive	414 (61%)		
Negative	257 (38%)		
Missing	10 (1%)		
ER			
Positive	462 (68%)		
Negative	198 (29%)		
Missing	21 (3%)		
C-erb			
Positive	405 (59%)		
Negative	114 (17%)		
Missing	162 (24%)		
Menopausal status			
Yes	321 (47%)		
No	247 (36%)		
Missing	113 (17%)		
Procreative times			
1	227 (33%)		
> 1	260 (38%)		
Missing	194 (29%)		
Age of menarche (years)			
≤ 14	340 (50%)		
> 14	233 (34%)		
Missing	108 (16%)		
BMI, kg/m^2^ (mean ± SD)^a^			
≤ 24	333 (49%)	240 (35%)	0.274
> 24	168 (25%)	114 (17%)	0.321
Missing	180 (26%)	326 (48%)	

^a^ Student’s t-test is used. *p* < 0.05 indicates statistical significance

*PR* progesterone receptor, *ER* estrogen receptor, *BMI* body mass index, *LN* lymph node

### Association between *CASC16* polymorphisms and BC risk

Five SNPs in the *CASC16* gene were selected and analysed in this case-control study. The distribution of allele frequencies between cases and controls was compared using chi-square test (Table 2). All five SNPs conformed to the HWE among controls (*p* > 0.05). It means appropriate SNP selection. And our results showed that the minor allele of two SNPs (rs4784227 and rs12922061) were significantly associated with increased BC susceptibility under allele model (OR = 1.22, 95% CI = 1.03–1.45, *p* = 0.022; OR = 1.21, 95% CI = 1.02–1.44, *p* = 0.026, respectively). We further examined the correlation between the genotypes of SNPs and BC risk by logistic regression analysis with adjustments for age under the codominant, dominant, recessive, and log-additive models (Table 3). We found that rs4784227 was related to a higher risk of BC in codominant model (T/C genotype, OR = 1.26, 95% CI = 1.00–1.57, *p* = 0.048), dominant model (T/C-T/T genotype, OR = 1.28, 95% CI = 1.03–1.59, *p* = 0.025) and the log-additive model (OR = 1.22, 95% CI = 1.03–1.45, *p* = 0.023). The rs12922061 also had a significant higher susceptibility to BC in codominant model (T/T genotype, OR = 1.63, 95% CI = 1.05–2.53, *p* = 0.030) and log-additive model (OR = 1.22, 95% CI = 1.03–1.45, *p* = 0.025). In contrast, rs3803662 was associated with a reduced risk of BC in recessive model (G/G genotype, OR = 0.69, 95% CI = 0.48–0.99, *p* = 0.042). Two SNPs (rs45544231 and rs3112612) were not observed association under any of the genetic models.

**Table 2:** The distribution of allele frequencies of *CASC16* SNPs in case and control

SNP ID	Alleles (minor/major)	Chromosome position	MAF		O (HET)	E (HET)	*p*^a^-HWE	OR (95% CI)	*p*^*b*^
			Case	Control					
rs3803662	G/A	chr16: 52552429	0.307	0.328	0.430	0.441	0.542	0.91 (0.77–1.06)	0.228
rs4784227	T/C	chr16: 52565276	0.277	0.239	0.356	0.363	0.596	1.22 (1.03–1.45)	**0.022**
rs45544231	C/G	chr16: 52598818	0.197	0.193	0.302	0.312	0.389	1.02 (0.85–1.24)	0.824
rs12922061	T/C	chr16: 52601088	0.285	0.247	0.385	0.372	0.410	1.21 (1.02–1.44)	**0.026**
rs3112612	G/A	chr16: 52601252	0.197	0.195	0.299	0.314	0.221	1.01 (0.84–1.23)	0.885

*SNP* single nucleotide polymorphisms, *MAF* minor allele frequency, *HWE* Hardy–Weinberg equilibrium

*p*^a^ values were calculated by exact test, *p*^a^ < 0.05 are excluded

*p*^*b*^ values were calculated by two–sided χ^2^, *p*^*b*^ < 0.05 indicates statistical significance

**Table 3:** Association between *CASC16* polymorphisms and breast cancer risk

SNP ID	Model	Genotype	Case N (%)	Control N (%)	OR (95% CI)	*p*^*a*^
rs3803662						
	Codominant	A/A	318 (46.70)	310 (45.66)	1	
		A/G	308 (45.23)	292 (43.00)	1.03 (0.82–1.29)	0.805
		G/G	55 (8.08)	77 (11.34)	0.70 (0.48–1.02)	0.061
	Dominant	A/A	318 (46.70)	310 (45.66)	1	
		A/G-G/G	363 (53.30)	369 (54.34)	0.96 (0.77–1.18)	0.700
	Recessive	A/A-A/G	626 (91.92)	602 (88.66)	1	
		G/G	55 (8.08)	77 (11.34)	0.69 (0.48–0.99)	**0.042**
	Log-additive	–	–	–	0.90 (0.77–1.06)	0.223
rs4784227						
	Codominant	C/C	353 (52.30)	394 (58.37)	1	
		T/C	270 (40.00)	240 (35.56)	1.26 (1.00–1.57)	**0.048**
		T/T	52 (7.70)	41 (6.07)	1.42 (0.92–2.18)	0.117
	Dominant	C/C	353 (52.30)	394 (58.37)	1	
		T/C-T/T	322 (47.70)	281 (41.63)	1.28 (1.03–1.59)	**0.025**
	Recessive	C/C-T/C	623 (92.30)	634 (93.93)	1	
		T/T	52 (7.70)	41 (6.07)	1.29 (0.84–1.97)	0.239
	Log-additive	–	–	–	1.22 (1.03–1.45)	**0.023**
rs45544231						
	Codominant	G/G	445 (65.35)	446 (65.59)	1	
		G/C	204 (29.96)	205 (30.15)	0.99 (0.79–1.26)	0.980
		C/C	32 (4.70)	29 (4.26)	1.11 (0.66–1.86)	0.707
	Dominant	G/G	445 (65.35)	446 (65.59)	1	
		G/C-C/C	236 (34.65)	234 (34.41)	1.01 (0.81–1.26)	0.928
	Recessive	G/G-G/C	649 (95.30)	651 (95.74)	1	
		C/C	32 (4.70)	29 (4.26)	1.11 (0.66–1.85)	0.701
	Log-additive	–	–	–	1.02 (0.85–1.23)	0.831
rs12922061						
	Codominant	C/C	348 (51.10)	381 (56.03)	1	
		C/T	278 (40.82)	262 (38.53)	1.16 (0.93–1.45)	0.187
		T/T	55 (8.08)	37 (5.44)	1.63 (1.05–2.53)	**0.030**
	Dominant	C/C	348 (51.10)	381 (56.03)	1	
		C/T-T/T	333 (48.90)	299 (43.97)	1.22 (0.99–1.51)	0.068
	Recessive	C/C-C/T	626 (91.92)	643 (94.56)	1	
		T/T	55 (8.08)	37 (5.44)	1.53 (0.99–2.35)	0.054
	Log-additive	–	–	–	1.22 (1.03–1.45)	**0.025**
rs3112612						
	Codominant	A/A	444 (65.29)	446 (65.59)	1	
		A/G	204 (30.00)	203 (29.85)	1.01 (0.80–1.28)	0.938
		G/G	32 (4.71)	31 (4.56)	1.04 (0.62–1.73)	0.891
	Dominant	A/A	444 (65.29)	446 (65.59)	1	
		A/G-G/G	236 (34.71)	234 (34.41)	1.01 (0.81–1.27)	0.911
	Recessive	A/A-A/G	648 (95.29)	649 (95.44)	1	
		G/G	32 (4.71)	31 (4.56)	1.03 (0.62–1.72)	0.899
	Log-additive	–	–	–	1.01 (0.84–1.22)	0.889

*CI* confidence interval, *OR* odds ratio, *SNP* single nucleotide polymorphism

^*a*^*p*-values were calculated by unconditional logistic regression analysis with adjustment for age

*p*^*a*^ < 0.05 indicates statistical significance

Highlighted in bold indicates the significant association between SNPs and breast cancer risk

### Stratified analyses between SNPs and BC risk based on age and clinical characteristics

The association between five SNPs and BC susceptibility was analyzed by logistic regression under age and clinical characteristic subgroups (Tables 4 and 5). On age-based stratification, rs4784227 would significantly increase risk of BC in allele model (OR = 1.34, 95% CI = 1.10–1.79, *p* = 0.007), codominant model (T/C genotype, OR = 1.46, 95% CI = 1.06–1.99, *p* = 0.019), dominant model (T/C-T/T genotype, OR = 1.51, 95% CI = 1.11–2.04, *p* = 0.008) and log-additive model (OR = 1.42, 95% CI = 1.10–1.82, *p* = 0.006) of the patients at age >  50 years (Table 4). And rs12922061 was also associated with an increased susceptibility to BC in allele model (OR = 1.36, 95% CI = 1.07–1.73, *p* = 0.012), codominant model (T/T genotype, OR = 1.91, 95% CI = 1.04–3.51, *p* = 0.036), dominant model (C/T-T/T genotype, OR = 1.41, 95% CI = 1.05–1.91, *p* = 0.024), and log-additive model (OR = 1.36, 95% CI = 1.07–1.73, *p* = 0.012) in subjects > 50 years old. However, the G/G genotype of rs3803662 played a reduced role in risk of breast cancer under the recessive model (OR = 0.53, 95% CI = 0.32–0.88, *p* = 0.014) of the patients ≤50 years. We also assessed the effect of *CASC16* gene polymorphisms on BC risk by clinical characteristics including clinical stage, tumor size, lymph node metastasis, and BMI. As was displayed in Table 5, it was found that T/T genotype of rs4784227 significantly improved risk of stage III/IV breast cancer patients (OR = 2.19, 95% CI = 1.08–4.46, *p* = 0.031) compared with stage I/II. The allele ‘C’ and C/C genotype of rs45544231, allele ‘G’ and G/G genotype of rs3112612 had protective effect on susceptibility of breast cancer with tumor size > 2 cm (OR = 0.72, *p* = 0.045; OR = 0.29, *p* = 0.001; OR = 0.71, *p* = 0.039; OR = 0.28, *p* = 0.001; respectively) than of tumor size ≤2 cm. The results further confirmed that TC + TT genotype of rs4784227 was significantly associated with an increased BC risk in lymph node metastasis positive individuals (OR = 1.41, 95% CI = 1.04–1.93, *p* = 0.028). Minor allele ‘T’ of rs12922061 was also noted to improve BC susceptibility in lymph node metastasis positive participants (OR = 1.30, 95% CI = 1.02–1.65, *p* = 0.034). In addition, the *CASC16* polymorphisms correlations with breast cancer were carried out in accordance with BMI-based stratification (Table 6). The results indicated that *CASC16* rs12922061 and rs4784227 polymorphisms were significantly correlated with increased risk of breast cancer in BMI >  24 kg/m^2^ subjects (T, OR = 1.54, 95% CI = 1.05–2.26, *p* = 0.026; TT genotype, OR = 13.41, 95% CI = 1.74–103.6, *p* = 0.013; T, OR = 1.49, 95% CI = 1.01–2.20, *p* = 0.042; respectively).

**Table 4:** The relationship of *CASC16* polymorphisms with breast cancer according to the stratification analysis by age

SNP	Model	Genotype	> 50 years				≤ 50 years			
			Case	Control	OR (95% CI)	*p*	Case	Control	OR (95% CI)	*p*
rs3803662	Allele	A	486 (70.43%)	464 (67.64%)	1		458 (68.15%)	448 (66.67%)	1	
		G	204 (29.57%)	222 (32.36%)	0.88 (0.70–1.10)	0.262	214(31.85%)	224(33.33%)	0.93 (0.74–1.17)	0.561
	Codominant	A/A	169 (48.99%)	151 (44.02%)	1		149 (44.34%)	159 (47.32%)	1	
		A/G	148 (42.90%)	162 (47.23%)	0.82 (0.60–1.12)	0.202	160 (47.62%)	130 (38.69%)	1.31 (0.95–1.81)	0.097
		G/G	28 (8.12%)	30 (8.75%)	0.83 (0.48–1.46)	0.526	27 (8.04%)	47 (13.99%)	0.61 (0.36–1.03)	0.063
	Dominant	A/A	169 (48.99%)	151 (44.02%)	1		149 (44.34%)	159 (47.32%)	1	
		A/G-G/G	176 (51.01%)	192 (55.98%)	0.82 (0.61–1.11)	0.191	187 (55.65%)	177 (52.68%)	1.13 (0.83–1.53)	0.443
	Recessive	A/A-A/G	317 (91.88%)	313 (91.25%)	1		309 (91.96%)	289 (86.01%)	1	
		G/G	28(8.12%)	30(8.75%)	0.92 (0.54–1.58)	0.768	27 (8.04%)	47 (13.99%)	0.53 (0.32–0.88)	**0.014**
	Log-additive	–	–	–	0.87 (0.69–1.10)	0.249	–	–	0.93 (0.74–1.17)	0.553
rs4784227	Allele	C	489 (71.28%)	531 (77.63%)	1		487 (73.34%)	497 (74.62%)	1	
		T	197 (28.72%)	153 (22.37%)	1.34 (1.10–1.79)	**0.007**	177 (26.66%)	169 (25.38%)	1.07 (0.84–1.37)	0.594
	Codominant	C/C	171 (49.85%)	205 (59.94%)	1		182 (54.82%)	189 (56.76%)	1	
		T/C	147 (42.86%)	121 (35.38%)	1.46 (1.06–1.99)	**0.019**	123 (37.05%)	119 (35.74%)	1.07 (0.78–1.49)	0.665
		T/T	25 (7.29%)	16 (4.68%)	1.88 (0.97–3.64)	0.061	27 (8.13%)	25 (7.51%)	1.13 (0.63–2.03)	0.678
	Dominant	C/C	171 (49.85%)	205 (59.94%)	1		182 (54.82%)	189 (56.76%)	1	
		T/C-T/T	172 (50.14)	137 (40.06%)	1.51 (1.11–2.04)	**0.008**	150 (45.18%)	144 (43.24%)	1.08 (0.80–1.47)	0.606
	Recessive	C/C-T/C	318 (92.71%)	326 (95.32%)	1		305 (91.87%)	308 (92.49%)	1	
		T/T	25 (7.29%)	16 (4.68%)	1.61 (0.84–3.07)	0.151	27 (8.13%)	25 (7.51%)	1.10 (0.62–1.94)	0.743
	Log-additive	–	–	–	1.42 (1.10–1.82)	**0.006**	–	–	1.07 (0.84–1.36)	0.589
rs45544231	Allele	G	569 (82.46%)	563 (81.83%)	1		525 (78.13%)	534 (79.46%)	1	
		C	121 (17.54%)	125 (18.17%)	0.96 (0.73–1.26)	0.759	147 (21.88%)	138 (20.54%)	1.08 (0.83–1.41)	0.548
	Codominant	G/G	239 (69.27%)	230 (66.86%)	1		206 (61.31%)	216 (64.29%)	1	
		G/C	91 (26.38%)	103 (29.94%)	0.85 (0.61–1.19)	0.341	113 (33.63%)	102 (30.36%)	1.16 (0.83–1.61)	0.377
		C/C	15 (4.35%)	11 (3.20%)	1.31 (0.59–2.92)	0.504	17 (5.06%)	18 (5.36%)	0.98 (0.49–1.96)	0.964
	Dominant	G/G	239 (69.27%)	230 (66.86%)	1		206 (61.31%)	216 (64.29%)	1	
		G/C-C/C	106 (30.72%)	114 (33.14%)	0.89 (0.65–1.23)	0.496	13 (38.69%)	120 (35.71%)	1.13 (0.83–1.55)	0.433
	Recessive	G/G-G/C	330 (95.65%)	333 (96.80%)	1		31 (94.94%)	318 (94.64%)	1	
		C/C	15 (4.35%)	11 (3.20%)	1.34 (0.62–3.04)	0.429	17 (5.06%)	18 (5.36%)	0.94 (0.47–1.85)	0.849
	Log-additive	–	–	–	0.96 (0.73–1.26)	0.763	–	–	1.08 (0.83–1.39)	0.568
rs12922061	Allele	C	485 (70.29%)	525 (76.31%)	1		48 (72.77%)	499 (74.26%)	1	
		T	205 (29.71%)	163 (23.69%)	1.36 (1.07–1.73)	**0.012**	18 (27.23%)	173 (25.74%)	1.08 (0.85–1.38)	0.537
	Codominant	C/C	171(49.57%)	200 (58.14%)	1		17 (52.68%)	181 (53.87%)	1	
		C/T	143 (41.45%)	125 (36.34%)	1.34 (0.98–1.83)	0.070	135(40.18%)	137 (40.77%)	1.01 (0.74–1.38)	0.953
		T/T	31 (8.99%)	19 (5.52%)	1.91 (1.04–3.51)	**0.036**	24 (7.14%)	18 (5.36%)	1.37 (0.72–2.61)	0.340
	Dominant	C/C	171 (49.57%)	200 (58.14%)	1		177 (52.68%)	181 (53.87%)	1	
		C/T-T/T	174 (50.43%)	144 (41.86%)	1.41 (1.05–1.91)	**0.024**	159 (47.32%)	155 (46.13%)	1.05 (0.78–1.42)	0.747
	Recessive	C/C-C/T	314 (91.01%)	325 (94.48%)	1		312 (92.86%)	318 (94.64%)	1	
		T/T	31 (8.99%)	19 (5.52%)	1.69 (0.94–3.06)	0.082	24 (7.14%)	18 (5.36%)	1.36 (0.73–2.56)	0.335
	Log-additive	–	–	–	1.36 (1.07–1.73)	**0.012**	–	–	1.09 (0.85–1.39)	0.518
rs3112612	Allele	A	569 (82.46%)	562(81.69%)	1		523 (78.06%)	533 (79.32%)	1	
		G	121(17.54%)	126(18.31%)	0.95 (0.72–1.25)	0.707	147 (21.94%)	139 (20.68%)	1.08 (0.83–1.40)	0.574
	Codominant	A/A	239 (69.28%)	230 (66.86%)	1		205 (61.19%)	216 (64.29%)	1	
		A/G	91 (26.38%)	102 (29.65%)	0.86 (0.61–1.20)	0.372	113 (33.73%)	101 (30.06%)	1.18 (0.85–1.64)	0.331
		G/G	15 (4.35%)	12 (3.49%)	1.20 (0.55–2.63)	0.641	17 (5.07%)	19 (5.65%)	0.94 (0.47–1.85)	0.848
	Dominant	A/A	239 (69.28%)	230 (66.86%)	1		205 (61.19%)	216 (64.29%)	1	
		A/G-G/G	106 (30.72%)	114 (33.14%)	0.89 (0.65–1.23)	0.496	130 (38.81%)	120 (35.71%)	1.14 (0.83–1.56)	0.415
	Recessive	A/A-A/G	330 (95.65%)	332 (96.51%)	1		318 (94.93%)	317 (94.35%)	1	
		G/G	15 (4.35%)	12 (3.49%)	1.26 (0.58–2.73)	0.560	17 (5.07%)	19 (5.65%)	0.88 (0.45–1.74)	0.722
	Log-additive	–	–	–	0.95 (0.73–1.24)	0.713	–	–	1.07 (0.83–1.38)	0.596

*CI* confidence interval, *OR* odds ratio, *SNP* single nucleotide polymorphism

*p* values were calculated by unconditional logistic regression adjusted by age; *p* < 0.05 indicates statistical significance

Highlighted in bold indicates the significant association between SNPs and breast cancer risk

**Table 5:** Correlations between *CASC16* polymorphisms and clinical characteristics of patients with breast cancer (adjusted by age)

SNP	Genotype	Clinical stage			Tumor size (cm)			LN metastasis		
		III,IV/I,II	OR (95% CI)	*p*-value	> 2 / ≤2	OR (95% CI)	*p*-value	Positive/Negative	OR (95% CI)	*p*-value
rs3803662	A	213/447	1		568/191	1		454/450	1	
	G	87/195	0.94 (0.69–1.27)	0.668	250/87	0.97 (0.72–1.30)	0.819	192/212	0.90 (0.71–1.14)	0.368
	AA	71/151	1		191/67	1		153/149	1	
	GA	71/145	1.02 (0.68–1.53)	0.917	186/57	1.13 (0.0.75–1.71)	0.546	148/152	0.95 (0.69–1.31)	0.744
	GG	8/25	0.66 (0.28–1.53)	0.331	32/15	0.73 (0.37–1.44)	0.367	22/30	0.71 (0.39–1.30)	0.270
	GA + GG	79/170	0.97 (0.66–1.43)	0.869	218/72	1.05 (0.71–1.55)	0.803	170/182	0.91 (0.67–1.24)	0.547
rs4784227	C	206/479	1		589/200	1		447/491	1	
	T	94/163	1.34 (0.99–1.81)	0.056	219/76	0.98 (0.72–1.33)	0.889	197/161	1.34 (1.05–1.72)	0.018
	CC	73/177	1		220/69	1		155/185	1	
	TC	60/125	1.19 (0.79–1.81)	0.400	149/62	0.77 (0.51–1.15)	0.194	137/121	1.35 (0.98–1.87)	0.069
	TT	17/19	2.19 (1.08–4.46)	**0.031**	35/7	1.59 (0.67–3.73)	0.292	30/20	1.79 (0.98–3.28)	0.059
	TC + TT	77/144	1.33 (0.90–1.96)	0.155	184/69	0.85 (0.58–1.25)	0.410	167/141	1.41 (1.04–1.93)	**0.028**
rs45544231	G	245/532	1		661/209	1		526/522	1	
	C	55/110	1.09 (0.76–1.55)	0.652	157/69	0.72 (0.52–0.99)	**0.045**	120/140	0.85 (0.65–1.12)	0.244
	GG	100/223	1		267/86	1		215/211	1	
	CG	45/86	1.14 (0.74–1.76)	0.549	127/37	1.09 (0.70–1.70)	0.693	96/100	0.94 (0.67–1.32)	0.726
	CC	5/120	0.89 (0.30–2.61)	0.833	15/16	0.29 (0.14–0.61)	**0.001**	12/20	0.59 (0.28–1.23)	0.160
	CG + CC	50/206	1.11 (0.73–1.69)	0.621	142/53	0.85 (0.57–1.27)	0.424	108/120	0.88 (0.64–1.22)	0.450
rs12922061	C	208/470	1		583/203	1		444/490	1	
	T	92/172	1.21 (0.89–1.63)	0.217	235/75	1.09 (0.80–1.48)	0.576	202/172	1.30 (1.02–1.65)	**0.034**
	CC	73/172	1		212/72	1		152/181	1	
	TC	62/126	1.18 (0.78–1.78)	0.424	159/59	0.93 (0.62–1.34)	0.709	140/128	1.30 (0.94–1.80)	0.108
	TT	15/23	1.58 (0.78–3.20)	0.209	38/8	1.64 (0.73–3.68)	0.232	31/22	1.68 (0.93–3.02)	0.084
	TC + TT	77/149	1.24 (0.84–1.84)	0.275	197/67	1.24 (0.84–1.84)	0.955	171/150	1.34 (0.99–1.85)	0.051
rs3112612	A	245/532	1		661/207	1		526/520	1	
	G	55/110	1.09 (0.76–1.55)	0.652	157/69	0.71 (0.52–0.98)	**0.039**	120/140	0.85 (0.65–1.11)	0.233
	AA	100/223	1		267/85	1		215/210	1	
	GA	45/86	1.14 (0.74–1.76)	0.549	127/37	1.08 (0.69–1.68)	0.739	96/100	0.94 (0.67–1.31)	0.702
	GG	5/12	0.89 (0.30–2.61)	0.833	15/16	0.28 (0.14–0.60)	**0.001**	12/20	0.58 (0.28–1.23)	0.155
	GA + GG	50/98	1.11 (0.73–1.69)	0.621	142/53	0.84 (0.56–1.25)	0.386	108/120	0.88 (0.64–1.21)	0.429

*p* values were calculated by unconditional logistic regression adjusted by age; *p* < 0.05 indicates statistical significance

*LN* lymph node

Highlighted in bold indicates the significant association between SNPs and breast cancer risk

**Table 6:** The associations between *CASC16* polymorphisms and BMI of breast cancer patients (adjusted by age and BMI)

SNP	Genotype	> 24 kg/m^2^			≤ 24 kg/m^2^		
		Case/Control	OR (95% CI)	*p*	Case/Control	OR (95% CI)	*p*
rs3803662	A	231/144	1		462/313	1	
	G	105/84	0.78 (0.55–1.11)	0.167	204/165	0.84 (0.65–1.08)	0.165
	AA	76/47	1		159/101	1	
	GA	79/50	0.97 (0.58–1.61)	0.899	144/111	0.83 (0.59–1.19)	0.313
	GG	13/17	0.46 (0.20–1.04)	0.063	30/27	0.73 (0.41–1.30)	0.287
	GA + GG	92/67	0.84(0.52–1.36)	0.481	174/138	0.81 (0.58–1.14)	0.230
rs4784227	C	231/174	1		476/361	1	
	T	103/52	1.49 (1.01–2.20)	**0.042**	180/117	1.17 (0.89–1.53)	0.263
	CC	77/65	1		172/135	1	
	TC	77/44	1.49 (0.91–2.46)	0.115	132/91	1.13 (0.79–1.60)	0.503
	TT	13/4	2.64 (0.82–8.54)	0.104	24/13	1.45 (0.71–2.95)	0.308
	TC + TT	90/48	1.59(0.98–2.58)	0.059	156/104	1.17 (0.83–1.63)	0.366
rs45544231	G	271/181	1		534/383	1	
	C	65/47	0.92 (0.61–1.41)	0.711	132/97	0.98 (0.73–1.31)	0.871
	GG	111/72	1		217/154	1	
	CG	49/37	0.83 (0.49–1.40)	0.481	100/75	0.96 (0.67–1.38)	0.817
	CC	8/5	0.98 (0.31–3.15)	0.979	16/11	1.07 (0.48–2.38)	0.869
	CG + CC	57/42	0.85 (0.51–1.40)	0.516	116/86	0.97 (0.69–1.34)	0.873
rs12922061	C	229/175	1		478/353	1	
	T	107/53	1.54 (1.05–2.26)	**0.026**	188/127	1.09 (0.84–1.42)	0.508
	CC	78/62	1		166/129	1	
	TC	73/51	1.15 (0.70–1.88)	0.581	146/95	1.20 (0.85–1.70)	0.306
	TT	17/1	13.41 (1.74–103.6)	**0.013**	21/16	1.01 (0.51–2.02)	0.968
	TC + TT	90/52	1.39 (0.86–2.25)	0.178	167/111	1.17 (0.84–1.64)	0.351
rs3112612	A	271/181	1		532/382	1	
	G	65/47	0.92 (0.61–1.41)	0.711	132/98	0.97 (0.72–1.30)	0.823
	AA	111/72	1		216/154	1	
	GA	49/37	0.83 (0.49–1.40)	0.481	100/74	0.97 (0.68–1.40)	0.887
	GG	8/5	0.98 (0.31–3.15)	0.979	16/12	0.99 (0.47–2.18)	0.994
	GA + GG	57/42	0.85 (0.51–1.40)	0.516	116/86	0.98 (0.69–1.38)	0.896

*p* values were calculated by unconditional logistic regression adjusted by age and BMI; *p* < 0.05 indicates statistical significance

Highlighted in bold indicates the significant association between SNPs and breast cancer risk

### Haplotype analyses of *CASC16* polymorphisms and breast cancer risk

**Fig. 1:**
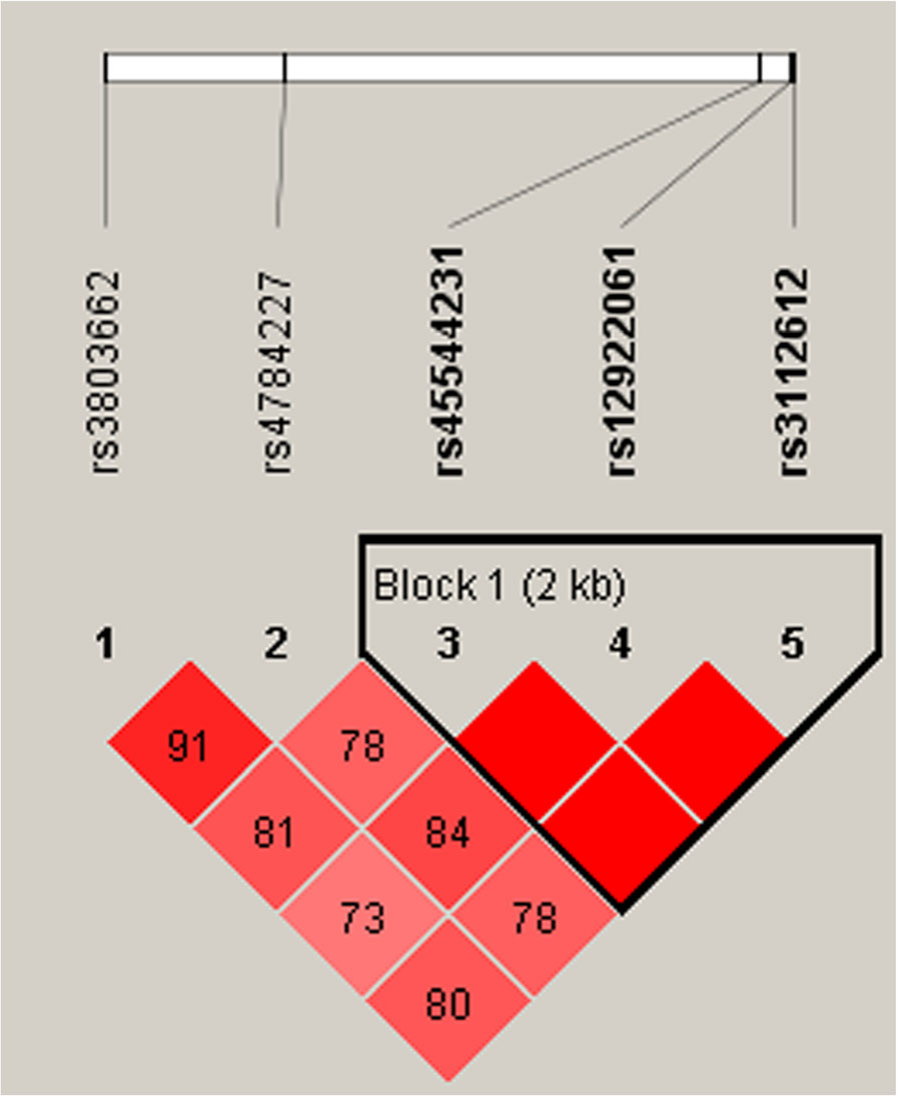
Haplotype block map for SNPs of *CASC16.* The numbers inside the diamonds indicate the D′ for pairwise analyses

We further examined the linkage disequilibrium (LD) and haplotype analyses of *CASC16* polymorphisms in case and control subjects via Haploview software and logistic regression. The LD plot was shown in Fig. 1, and LD block was consisted of three SNPs including rs45544231, rs12922061 and rs3112612. The haplotype analysis revealed that G_rs45544231_ T_rs12922061_ A_rs3112612_ and G_rs45544231_ C_rs12922061_ A_rs3112612_ haplotypes in the *CASC16* gene were found to reduce risk of breast cancer (OR = 0.82, 95% CI = 0.69–0.98, *p* = 0.025; OR = 0.85, 95% CI = 0.73–0.99, *p* = 0.039; respectively; Table 7).

**Table 7:** The haplotype frequencies of *CASC16* polymorphisms and their associations with breast cancer risk

SNP	Haplotype	Frequency		Without adjusted		With adjusted	
		Case	Control	OR (95% CI)	*p*	OR (95% CI)	*p*
rs45544231|rs12922061|rs3112612	CCG	0.80	0.81	0.98 (0.81–1.18)	0.827	0.98 (0.81–1.18)	0.831
rs45544231|rs12922061|rs3112612	GTA	0.72	0.75	0.82 (0.69–0.98)	0.025	0.82 (0.69–0.98)	**0.025**
rs45544231|rs12922061|rs3112612	GCA	0.52	0.56	0.85 (0.73–0.99)	0.039	0.85 (0.73–0.99)	**0.039**

*p* value calculated by Wald test with and without adjusted by age

Highlighted in bold indicates the significant association between SNPs and breast cancer risk

## Discussion

In the present case-control study, 681 breast cancer patients and 680 free-cancer subjects were recruited to evaluate the correlation between *CASC16* variants and BC risk in a Northwest Chinese female population. The research showed that *CASC16* polymorphisms (rs4784227, rs12922061, and rs3803662) were significantly associated with BC susceptibility. Furthermore, rs4784227, rs12922061, rs3803662, rs45544231, and rs3112612 polymorphisms were associated with breast cancer patients with stratified subgroups including age, lymph node metastasis status, clinical stage, tumor size, and BMI. Taken together, these findings suggested an important role for the *CASC16* gene in the occurrence of breast cancer.

Rs3803662 was identified SNP in the *CASC16* gene association with breast cancer as previously published studies (Udler et al. 2010). Considerably increased association between rs3803662 in the *CASC16* gene and breast cancer was studied in Japanese and Caucasian women (Low et al. 2013) (Guan et al. 2016). In contrast, our present study indicated that rs3803662 played a protective role in BC risk (OR = 0.69, *p* = 0.042) in a Northwest Chinese population, and the same finding was showed in patients ≤50 years (OR = 0.53, *p* = 0.014). However, Edward A et al. suggested that no relationship was found between rs3803662 and breast cancer in African-American population (Ruiz-Narvaez et al. 2010). The SNP rs12922061, located in the first intron of *LOC643714*, was identified as a susceptibility variant of breast cancer in a Japanese GWAS (Huang et al. 2019). In our study, rs12922061 polymorphism was associated with an increased susceptibility to BC or patients with lymph node metastasis, age ≤ 50 years and BMI > 24 kg/m^2^ individuals. Data from Chen’s research showed that the increased association only observed in BC patients, no significant association was found in stratified subgroups in Southeast China population (Chen et al. 2016b). In summary, these results may be due to the differences in geography, ethnicity, and region among population, which leads to genetic variants. Our study also indicated that rs3803662 and rs12922061 played crucial roles in the progression of breast cancer.

Rs447842227 polymorphism in *CASC16* is also a strong current candidate association with breast cancer risk. This study found that rs4784227 significantly increased susceptibility to breast cancer patients with age > 50 years, clinical stage III/IV, lymph node metastasis status, and BMI > 24 kg/m^2^. These findings were in line with that of He (2014) who confirmed that rs4784227 could increase risk of breast cancer in a Southern Chinese population, while they hadn’t identified correlation under stratified analysis (He et al. 2014) due to the difference in population. In a word, our present findings revealed that rs44842227 might be associated with age, clinical stage, lymph node metastasis status, and BMI in breast cancer.

Furthermore, our study firstly revealed that rs45544231 and rs3112612 in *CASC16* played protective roles in tumor size > 2 cm individuals. In addition, we also studied linkage disequilibrium (LD) and haplotype analyses of *CASC16* polymorphisms in cases and controls. Haplotype analyses disclosed that G_rs45544231_ T_rs12922061_ A_rs3112612_ and G_rs45544231_ C_rs12922061_ A_rs3112612_ haplotypes reduced BC risk.

The major limitation of this study was the fact that we just studied the association between *SCAC16* variants and breast cancer in a Northwest Chinese population. Further research in other areas or races in China is an essential step in supplementing the extant data. Besides, we determined the role of *CASC16* SNPs in risk of breast cancer but there were still not detecting function of *CASC16* in occurrence and evolution of breast cancer. Therefore, next work should focus on exploring the functions of *CASC16* in breast cancer. In spite of its limitations, the study certainly adds to our understanding of the association between SNP variants and breast cancer. Moreover, our present work provided the possibility of using these SNPs to diagnose breast cancer in the future.

## Conclusions

In summary, *CASC16* rs4784227 and rs12922061 were significantly related to increased susceptibility to breast cancer. Stratification analysis revealed that rs4784227 and rs12922061 would increase BC susceptibility in age > 50 years. Rs3803662 was a reduced factor of BC in age ≤ 50 years. Rs4784227 was significantly improved susceptibility to BC patients in stage III/IV. The rs45544231 and rs3112612 had protective effects on BC with tumor size > 2 cm. Rs4784227 and rs12922061 could increase BC risk in lymph node metastasis positive individuals. *CASC16* rs12922061 and rs4784227 polymorphisms were correlated with increased BC risk in BMI > 24 kg/m^2^. We noted that G_rs45544231_ T_rs12922061_ A_rs3112612_ and G_rs45544231_ C_rs12922061_ A_rs3112612_ haplotypes reduced BC risk. These findings would give some new insights in the molecular mechanism of breast cancer occurrence.
